# Water Deprivation and Sowing Times Alter Plant–Pollination Interactions and Seed Yield in Sunflower, *Helianthus annuus* L. (Asteraceae)

**DOI:** 10.3390/plants13223194

**Published:** 2024-11-14

**Authors:** Qasim Ali, Mudssar Ali, Fawad Zafar Ahmad Khan, Ahmed Noureldeen, Akram Alghamdi, Hadeer Darwish, Akash Fatima, Ahmad Ibrahim Jalali, Kit Prendergast, Shafqat Saeed

**Affiliations:** 1 Institute of Plant Protection, Muhammad Nawaz Shareef University of Agriculture Multan, Multan 60000, Pakistan; 2 Department of Outreach and Continuing Education, Muhammad Nawaz Shareef University of Agriculture Multan, Multan 60000, Pakistan; 3 Department of Biology, College of Sciences, Taif University, P.O. Box 11099, Taif 21944, Saudi Arabia; 4 Department of Biotechnology, College of Sciences, Taif University, P.O. Box 11099, Taif 21944, Saudi Arabia; 5 Institute of Plant Breeding & Biotechnology, Muhammad Nawaz Shareef University of Agriculture Multan, Multan 60000, Pakistan; 6 Institute for Life & the Environment, University of Southern Queensland, Toowoomba, QLD 4350, Australia

**Keywords:** temperature, water deprivation, sunflower, plant-pollinator interactions, seed production, yield

## Abstract

Climate change effects, including temperature extremes and water stress, cause abiotic stress in plants. These changes directly affect flowering and the flower reward system for pollinators, influencing plant–pollinator interactions and ultimately seed production in flowering plants. Here, we tested the effects of water deprivation on the behavior of various pollinator species, plant–pollinator interactions, and the seed yield of sunflower, *Helianthus annuus* L. (Asteraceae). Sunflower was sown during four different months (January–April) and subjected to two different water availability levels (well-watered and water-deprived). Pollinator abundance was recorded five times a day (8:00 am, 10:00 am, 12:00 pm, 2:00 pm, and 4:00 pm) from flower heads and the florets. In addition, foraging behavior was also recorded. We found that lowest abundance, visit duration, and visitation rate occurred in April-sown sunflower. The European honey bee *Apis mellifera* L. (Hymenoptera: Apidae) was the most abundant visitor to sunflower, the hover fly *Eristalinus aeneus* (Diptera: Syrphidae) exhibited the longest visit duration, while *Xylocopa* sp. (Hymenoptera: Apidae) exhibited the highest visitation rate. The visitation rate of bees was significantly affected by water stress, with more bee visits occurring under well-watered conditions. Additionally, plant parameters, including flower head diameter, head weight, seed number, and seed weight, were significantly lower in the water-deprived treatments in April-sown sunflower. Open flowers without the pollination exclusion cages showed a higher yield, indicating the pollination dependence of sunflower. In conclusion, the plant modifications induced by sowing months and water-deprived conditions may alter pollinator behavior and may ultimately affect sunflower yield.

## 1. Introduction

Climate change is increasing the amount and intensity of extreme heat, as well as reducing rainfall events [1,2]. With rising temperatures and uneven rainfall patterns, areas with low rainfall are expected to be at a higher risk of drought [3]. The duration of drought events is also expected to increase as global warming is causing a higher rate of soil moisture evaporation, and hence, reducing water availability to plants [4,5,6]. As a result, plants growing under drought stress experience changes in turgor, stomatal conductance, growth, and photosynthetic activity [7,8]. The water stress alters the activity of plant-dependent arthropods, including herbivores and pollinators [9,10,11]. Therefore, the study of plant–pollinator mutualism is crucial in the context of the increasing threat of drought. The study of plant–pollinator mutualisms is especially important because these interactions are responsible for food production [12,13,14].

Multiple studies have reported that decreased seed production in water-stressed plants might result from both the direct effects of water stress and altered behavior of insect pollinators [15,16]. Water stress affects floral traits, leading to changes in pollinator behavior and visitation [17,18]. Water stress can also directly affect flower parameters important for attracting pollinators, such as size, number, color, nectar volume, and pollen quality [19]. Similarly, it can also alter the release of volatile organic compounds that insect pollinators use to locate flowers [20]. Such changes in floral emissions can negatively affect pollinator visitation, and hence reduce plant reproductive success.

For example, a study on borage, *Borago officinalis* L. (Boraginaceae), under two water regimes (well-watered and water-stressed conditions) and three temperature levels reported decreased flower number, corolla surface, nectar sugar content, and pollen viability in water-stressed plants, resulting in bumblebee visitation being reduced by almost 50% to the water-stressed flowers [21]. A mesocosm study concluded that water stress leads to a decline in floral resources available for pollinators by reducing flowering intensity, flower numbers, and nectar sugars [22]. Another study found that the corolla size, nectar volume, and pollinator visits were highest on plants with optimum water availability, and plants with higher water supply in turn had a higher seed set [21].

Reduced seed set in water-stressed plants may also result from the combined effects of plant physiological changes and indirect changes in pollinator behavior, which can vary with pollinator species, e.g., generalist vs. specialist pollinators [20,23]. To understand the direct and indirect effects of water stress on plant–pollinator interactions, it is crucial to study the pollinator species, pollinator behavior, and the plant’s reproductive parameters. 

Sunflowers are known to be sensitive to climatic conditions [24], but it is still unclear whether this sensitivity affects pollinator visitation. Water availability may affect key aspects of pollinator visits that influence pollination, including the type of pollinators visiting flowers, visitation frequency, and stay time. Pollinator assemblages play a great role in crop production because they influence the probability of pollination success. Different pollinator species vary in their effectiveness, and a diverse assemblage of pollinators enhances pollination, leading to better crop yields. Research to date shows that sunflower benefits from insect pollination [25], that different insects vary in their effectiveness as sunflower visitors [26,27], that a diversity of visitors enhances pollination [28], and that the assemblages of pollinator species contribute to these enhanced pollination services [29]. Greater visitation rates also lead to increased seed yield in sunflower [29]. Visit duration, i.e., how long an insect visits a flower, can also benefit pollination [30]; however, this may not be important in the case of sunflower [29].

In the current study, we tested how pollinator abundance and taxon, behavior, and crop yield fluctuated in sunflower grown across different sowing months and under two water regimes. We tested four predictions: (1) water stress will negatively affect the abundance and diversity of pollinators. To test this prediction, we measured the abundance and diversity of pollinators visiting sunflower sown in four different months, under two water levels; (2) water stress will negatively affect pollinator behavior, in terms of visit duration and visitation rate. To test the second prediction, we quantified the visit duration and visitation rate of different bee species on both flower heads and florets of sunflower; (3) water stress will negatively affect yield parameters (including head diameter, head weight, seed number, and seed weight) of sunflower. We tested this prediction by measuring plant traits and seed parameters of sunflower grown in both well-watered and water-stressed plots; and (4) open and caged pollination treatments will affect yield parameters (including head diameter, head weight, seed number, and seed weight) of sunflower. This final prediction was tested by maintaining two sets of flower heads, one open to provide access to pollinators and the other without pollinators. Our experiment is the first evidence providing a detailed overview of the effect of water stress on pollinator behavior and yield parameters in sunflower.

## 2. Materials and Methods

### 2.1. Study Area

The study was performed at the experimental farm of Muhammad Nawaz Shareef University of Agriculture Multan, Pakistan (30°08′48″ N 71°26′55″ E). Sunflower hybrid cultivar (Hysun 33, Syngenta, Karachi, Pakistan) was sown over an area of one acre (with an area of 1000 m^2^ dedicated for each sowing date) in four different months (middle of January, February, March, and May). Sunflower was sown at four different times as the planting dates of the crops are changing due to current temperature fluctuations in Pakistan. This is a male-fertile cultivar, and sunflower has male and female flowers on the same plant. Nearby vegetation included crops like sorghum *Sorghum bicolor* (L.) Moench (Poaceae), upland cotton *Gossypium hirsutum* L. (Malvaceae), cucumber *Cucumis sativus* L. (Cucurbitaceae), and perennial trees including Indian rosewood *Dalbergia sissoo* Roxb. (Fabaceae) and gum arabic tree (*Vachellia nilotica* (L.) PJH Hurter & Mabb (Fabaceae). Multan is a tropical desert, and the environmental conditions are characterized by cold winters and an intense summer season. The average temperature in summer is 40 ± 5 °C and in winter it remains 10 ± 5 °C; moreover, the annual rainfall ranges from 127 to 254 mm [31].

### 2.2. Field Layout

For each sowing date, the designated area was divided into two smaller blocks: one well-watered and one water-deprived. Each block measured 16.8 m × 7.3 m (L × W) and was replicated three times. A distance of 10 m was maintained between each block. A total of five irrigations were applied for the well-watered treatment and two for the water-deprived treatment. Both treatments were replicated three times. A space of 3 m was maintained between each block [32]. At the flower budding stage, we stopped irrigating the water deprivation block. The first irrigation was applied 20 days after seed sowing. The second irrigation was applied 20 days after the first. The third was at sunflower head formation, the fourth at grain formation, and the final irrigation during the milking stage of seeds. Standard agronomic practices were implemented for weed management, and plant bottoms were dug up to prevent plant lodging. Integrated pest management strategies were employed to control the pest attack. A soil moisture meter was used to measure water content from five different locations in the field for soil moisture measurement. Space within the plots was left for pollinators to move freely and perform pollination services.

### 2.3. Pollinator Abundance and Behavior Evaluation

Pollinator abundance data were collected five times a day (8:00 am, 10:00 am, 12:00 pm, 2:00 pm, and 4:00 pm), along with relative humidity, wind speed, and temperature. The data were collected from the January-sown crop, which flowered from 1–22 April; the February-sown crop, which flowered from 18 April to 5 May; the March-sown crop, which flowered from 12–28 May; and the April-sown crop, which flowered from 10–25 June. The data were collected after every three days. For each observation of pollinator abundance, 15 sunflower heads (capitulum heads) were randomly selected from each block, and data from all flower visitors were recorded by an expert for one minute for each flower. The expert had previous experience in bee identification and behavior experiments. Data were recorded when flowers were 10% open. Insect foraging behavior, including the number of flowers visited by an insect pollinator in one minute (visitation rate/flower), number of florets visited by an insect pollinator in one minute (visitation rate/floret), and the time spent by each pollinator species on one flower (visit duration), was recorded.

### 2.4. Pollination Treatments

To measure differences in seed production due to pollinating insects, we maintained thirty plants in cages (self-pollination). These plants were covered with muslin cloth bags at the bud stage to prevent visits by insect pollinators. Additionally, thirty plants were open-pollinated (allowing pollinator interaction with the capitulum heads). Both caged and open-pollinated plants were maintained for comparison in well-watered and water-deprived treatments. Pollinator abundance, visit duration, and visitation rate were recorded under different water regimes and sunflower crop sowing times. The comparison of different water levels was evaluated by monitoring bee pollinator activity.

### 2.5. Harvesting

Harvesting time varied based on the sowing time. Crop harvesting was conducted in the morning when the seed heads and plants were completely dry in each plot. Thirty sunflower heads were randomly harvested from each treatment (water-deprived and well-watered), and this procedure was consistent for each sowing time (January, February, March, and April).

### 2.6. Yield Parameters

Sunflower heads were harvested from various treatments and then taken to the laboratory to gather data on various yield factors of sunflower, including head diameter, head weight, number of seeds per head, and seed weight per head. Sixty heads (30 from open and 30 from caged) of sunflower were harvested from each plot (well-watered and water-deprived) and their diameters were measured in inches. The weight of sunflower heads was measured using a weight balance for all treatments. After measuring the head diameter and weight, the total number of seeds per head was determined for all treatments. The seed weight was measured using a digital weight balance after separating the seeds per flower head. For this purpose, thirty sunflower heads were harvested from each treatment.

### 2.7. Statistical Analysis

The data were analyzed using analysis of variance (ANOVA) (2-way interaction) to compare pollinator abundance, visitation rate, and visit duration, as well as sunflower yield parameters including head diameter, head weight, number of seeds per head, and seed weight per head across different treatments. Pairwise comparisons within treatments were performed using the least significant difference (LSD) test at a significance level of α = 0.05.

## 3. Results

**Figure 1:**
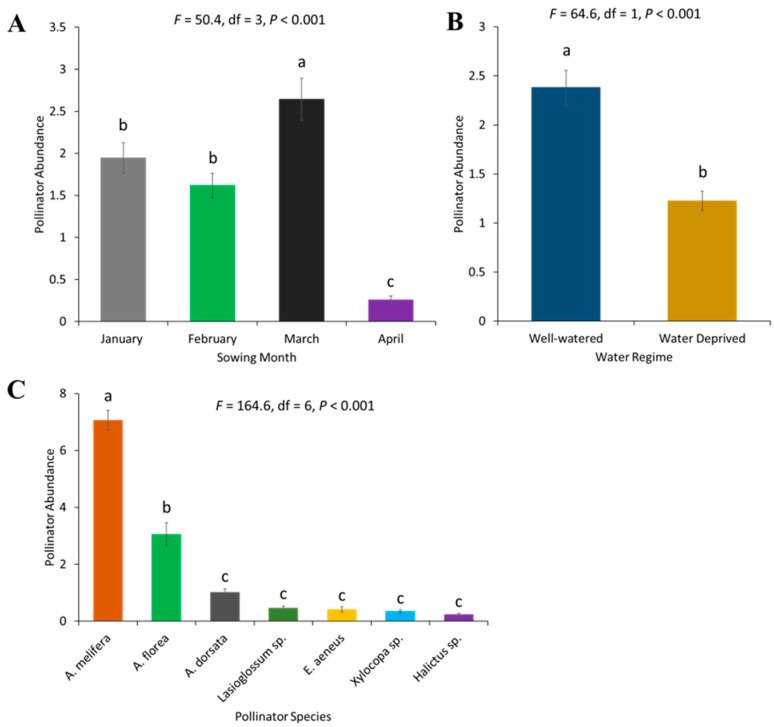
Pollinator abundance on sunflower: (**A**) sowing months of the crop, (**B**) water regimes, (**C**) bee pollinators. Error bars represent the standard error of the mean (SEM) across replicates. Different letters indicate significant differences between treatments based on the LSD test at a 5% significance level.

Overall, in all the treatments, the number of pollinators, predominantly bees, visiting the sunflower significantly differed between different sowing months (*F* = 50.42, df = 3, *p* < 0.001). A higher pollinator abundance was recorded in March, followed by January and February-sown sunflower, while the lowest number was recorded in April (Figure 1A). The abundance of pollinators also varied significantly between watering treatments (*F* = 64.62, df = 1, *p* < 0.001), with the highest abundance recorded in the well-watered treatment (Figure 1B). In all the treatments, a total of seven pollinator species visited sunflower. There were significant differences in the abundance of each species (*F* = 164.59, df = 6, *p* < 0.001) (Figure 1C). *A. mellifera* was the most frequent visitor, followed by *A. florea*, while the numbers of all other pollinators, including *A. dorsata*, *Lasioglossum* sp., *Eristalinus aenus*, *Xylocopa* sp. and *Halictus* sp., were not statistically different. 

**Figure 2:**
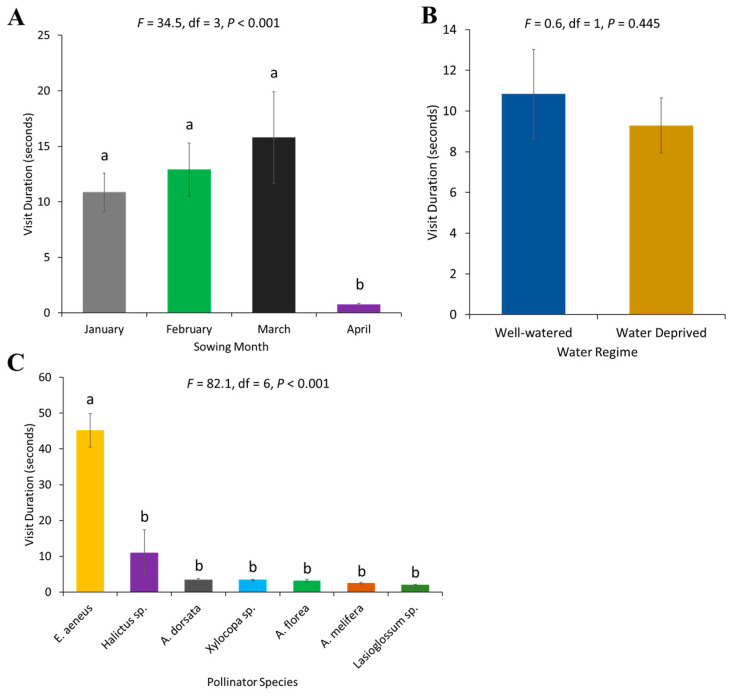
Visit duration of pollinators on sunflower: (**A**) sowing months of the crop, (**B**) water regimes, and (**C**) pollinator species. Error bars represent the standard error of the mean (SEM) across replicates. Different letters indicate significant differences between treatments based on the LSD test at a 5% significance level.

The average visit duration of bees was significantly different in sunflower sown across different months (*F* = 34.54, df = 3, *p* < 0.001). The lowest visit duration was observed for the April-sown sunflower (Figure 2A). Visit duration did not differ between watering regimes (*F* = 0.58, df = 1, *p* = 0.4453) (Figure 2B). Visit duration differed according to pollinator species (*F* = 82.12, df = 6, *p* < 0.001), with the longest visit duration recorded for *E. aeneus*, followed by all other species (Figure 2C). 

**Figure 3:**
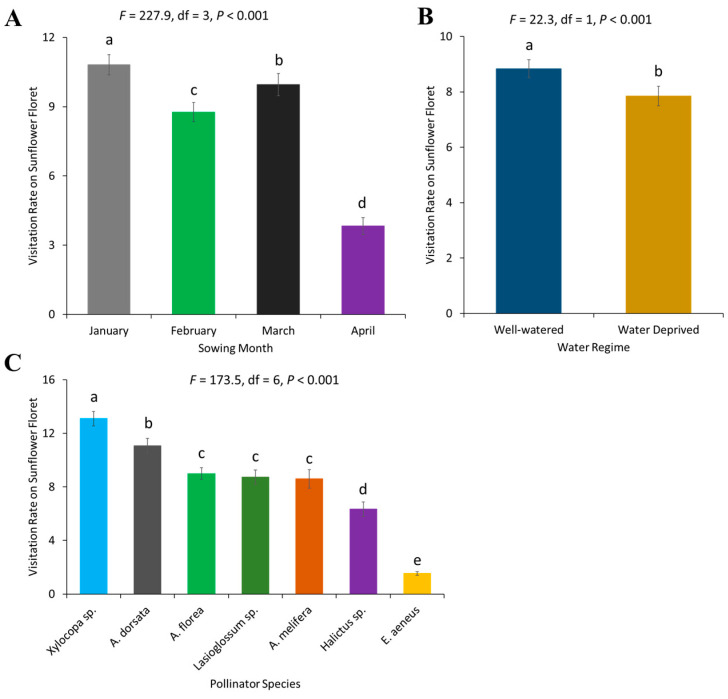
Visitation rate of pollinators on sunflower floret: (**A**) sowing months of the crop, (**B**) water regimes, and (**C**) pollinator species. Error bars represent the standard error of the mean (SEM) across replicates. Different letters indicate significant differences between treatments based on the LSD test at a 5% significance level.

The visitation rate of pollinators on sunflower florets differed between months (*F* = 227.99, df = 3, *p* < 0.001). The highest visitation rate of pollinators was recorded in January, followed by March and February, while the visitation rate was lowest on the flowers of the April-sown crop (Figure 3A). The visitation rate of pollinators was significantly higher on well-watered plots as compared to the experimental water-deprived plots (*F* = 22.26, df = 1, *p* < 0.001) (Figure 3B). Moreover, the average visitation rate of individual bee species on sunflower florets also varied significantly (*F* = 173.48, df = 6, *p* < 0.001). *Xylocopa* sp. was the most frequent visitor and *E. aeneus* was least frequent (Figure 3C).

**Figure 4:**
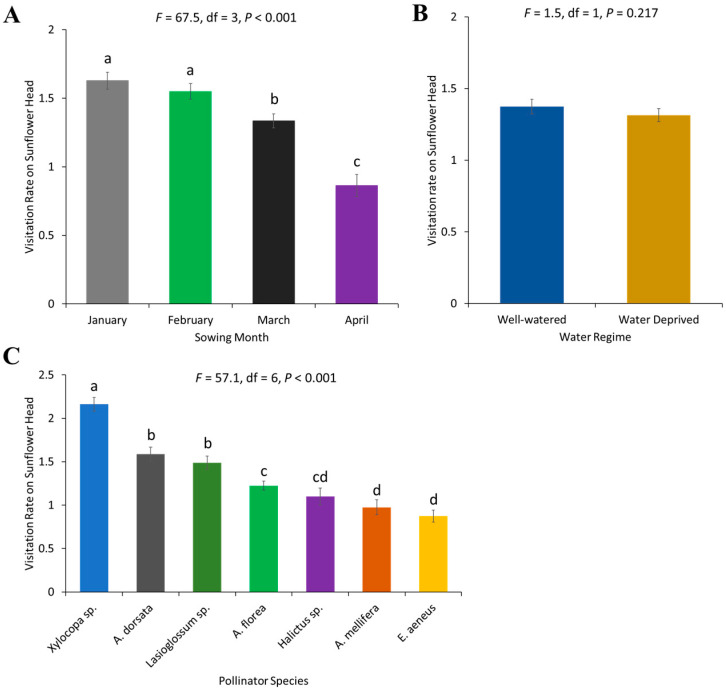
Visitation rate of pollinators on sunflower head: (**A**) sowing months of the crop, (**B**) water regimes, and (**C**) pollinator species. Error bars represent the standard error of the mean (SEM) across replicates. Different letters indicate significant differences between treatments based on the LSD test at a 5% significance level.

The visitation rate of pollinators on sunflower heads varied by month of sowing (*F* = 67.51, df = 3, *p* < 0.001), with a higher visitation rate recorded for the crop sown in January and February, while the lowest visitation rate was observed for April (Figure 4A). However, no statistical differences were found for the visitation rate on sunflower heads according to water regimes (*F* = 1.53, df = 3, *p* = 0.2171) (Figure 4B). The visitation rate of individual bees was significantly different (*F* = 57.06, df = 6, *p* < 0.001), with *Xylocopa* sp. being the most frequent visitor on flower heads (Figure 4C).

**Figure 5:**
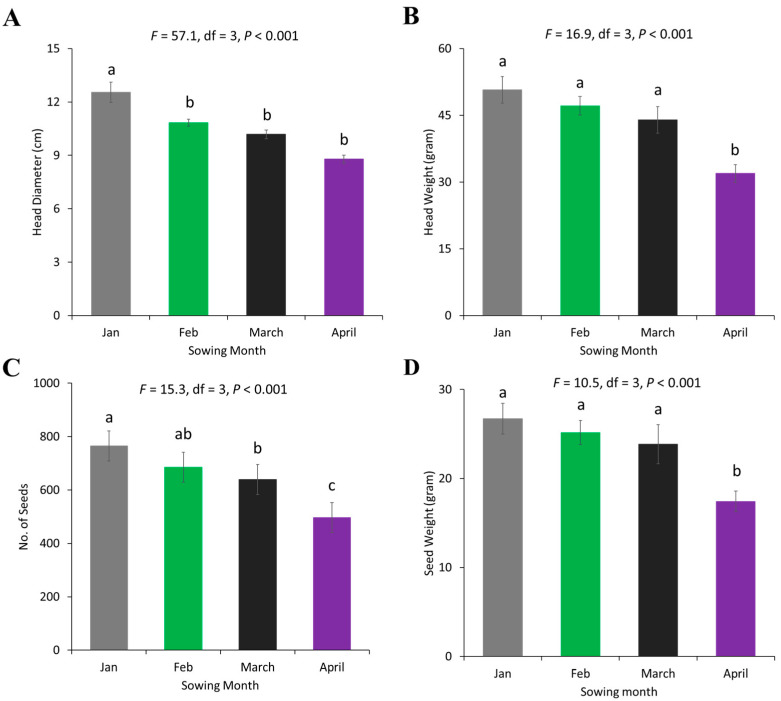
Yield parameters of sunflower in different sowing months, (**A**) head diameter, (**B**) head weight, (**C**) seed number, and (**D**) seed weight. Error bars represent the standard error of the mean (SEM) across replicates. Different letters indicate significant differences between treatments based on the LSD test at a 5% significance level.

**Figure 6:**
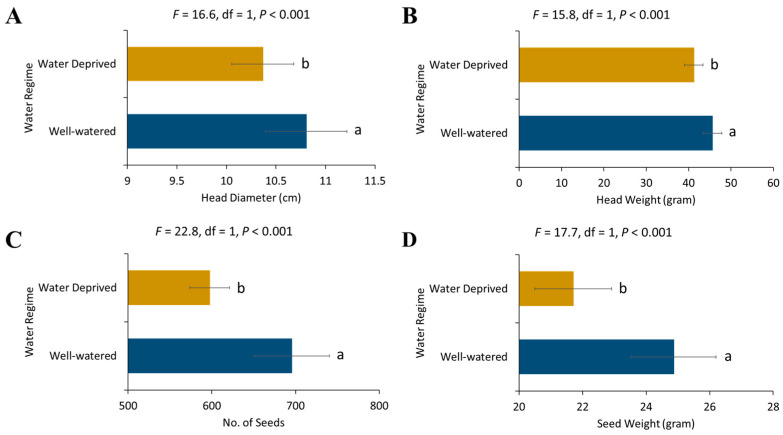
Effect of water regimes on different yield parameters of sunflower (**A**) head diameter, (**B**) head weight, (**C**) seed number, and (**D**) seed weight. Error bars represent the standard error of the mean (SEM) across replicates. Different letters indicate significant differences between treatments.

For the sunflower yield parameters, significant differences were observed for head diameter (*F* = 57.08, df = 3, *p* < 0.001), head weight (*F* = 16.97, df = 3, *p* < 0.001), seed number (*F* = 15.30, df = 3, *p* < 0.001) and seed weight (*F* = 10.50, df = 3, *p* < 0.001) for sunflower grown in different months. The sunflower head diameter, head weight, seed number, and seed weight were significantly greater in January-sown sunflower as compared to April-sown sunflower (Figure 5). Yield parameters also varied significantly between water regimes. The head diameter (*F* = 16.61, df = 1, *p* = 0.0003), head weight (*F* = 15.79, df = 1, *p* = 0.0003), seed number (*F* = 22.82, df = 1, *p* < 0.001) and seed weight (*F* = 17.68, df = 1, *p* = 0.0002) were significantly higher in well-watered sunflowers as compared to water-deprived ones (Figure 6).

**Figure 7:**
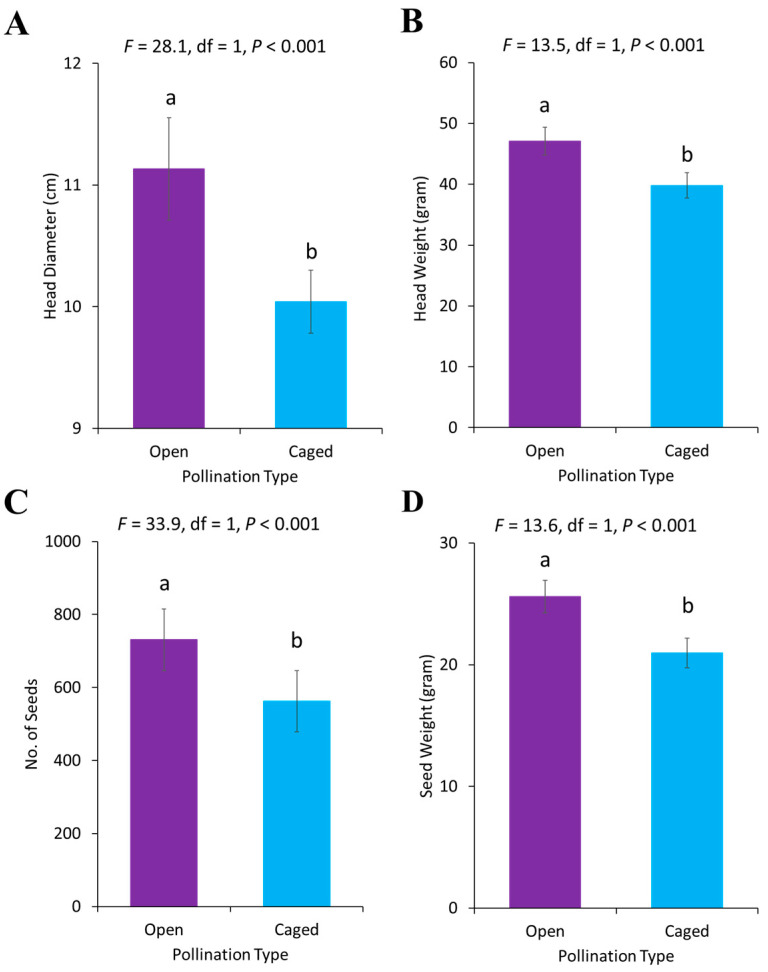
Effect of pollination types on different yield parameters of sunflower (**A**) head diameter, (**B**) head weight, (**C**) seed number, (**D**) seed weight. Error bars represent the standard error of the mean (SEM) across replicates. Different letters indicate significant differences between treatments.

For different pollination treatments, yield parameters were significantly different, i.e., head diameter (*F* = 28.05, df = 1, *p* < 0.001), head weight (*F* = 13.55, df = 1, *p* = 0.0008), seed number (*F* = 33.94, df = 1, *p* < 0.001) and seed weight (*F* = 13.60, df = 1, *p* = 0.0008). A significantly higher yield was observed in open pollinated treatments (with pollinators interacting with flowers) as compared to the caged treatments (pollination hindered using mesh net) (Figure 7).

We observed significant differences for the combined effect of sowing months and water regimes on the abundance of insect pollinators (Table 1). The highest abundance of insect pollinators was recorded in well-watered plots of the March-sown crop. The lowest pollinator abundance was observed for both water-deprived and well-watered plots of the April-sown crop (Table 1). In January and February, the abundance of *A. mellifera* was significantly higher than all other pollinators, while in March *A. mellifera* and *A. florea* were significantly higher. The highest rate of pollinator visitation on sunflower florets was observed in the well-watered treatment of January and March, while the lowest was observed for the well-watered and water-deprived treatments of April (Table 1). No significant differences were observed for the visit duration of pollinators in different water regimes (Table 1).

The species-specific abundance, visit duration, visitation rate per flower, and visitation rate per floret showed significant differences (Table 2). For pollinator abundance, *A. mellifera* was significantly higher in the months of January, February, and March. *A. florea* was significantly more abundant in the month of March compared with the other months. The visit duration of *E. aeneus* was significantly greater in the sunflower sown in January, February, and March (Table 2). The visitation rate of *Xylocopa* sp. and *A. dorsata* per floret was significantly greater in January and March, respectively, as compared to all other bees. The visitation rate (per floret) of *Xylocopa* sp. in January and *A. dorsata* in March was significantly higher as compared to other species (Table 2). For the flower head, the visitation rate of *Xylocopa* sp. was significantly higher in January, February, and April-sown sunflower compared with March. *A. dorsata* had a significantly higher visitation rate in January-sown sunflower compared with the other months (Table 2).

Significant differences were found for the abundance of different species (*F* = 16.68, df = 6, *p* < 0.001), visit duration of species (*F* = 2.89, df = 6, *p* = 0.0089) and visitation rate of each species on a floret (*F* = 2.72, df = 6, *p* = 0.0129), while no differences were recorded for the visitation rate of different species on the flower head (*F* = 1.69, df = 6, *p* = 0.1216). *A. mellifera* abundance was highest in the flowers in well-watered plots. The visit duration of *E. aeneus* was significantly higher than all other pollinators in both well-watered and water-deprived regimes (Table 3). The visitation rate of *Xylocopa* sp. on every floret was significantly higher than all other pollinators (Table 3).

No significant interactive effects of the sowing months and water regime were observed in flower head diameter (*F* = 1.42, df = 3, *p* = 0.25), head weight (*F* = 0.87, df = 3, *p* = 0.46), seed number (*F* = 1.04, df = 3, *p* = 0.38) and seed weight (*F* = 1.02, df = 3, *p* = 0.39) (Table 4). Similarly, no significant interactive effects of the water regime and pollination treatments were observed on flower head diameter (*F* = 0.13, df = 1, *p* = 0.7176), head weight (*F* = 1.55, df = 1, *p* = 0.0008), seed number (*F* = 13.55, df = 1, *p* = 0.0008) and seed weight (*F* = 13.55, df = 1, *p* = 0.0008) (Table 5). On the other hand, significant differences were observed in the two-way interaction of the sowing month and pollination treatments on plant parameters (Table 6). A significantly greater head diameter and higher seed number were observed in the open-pollinated flowers of the January-sown crop (Table 6).

## 4. Discussion

The current study reports changes in pollinator behavior and plant yield parameters due to water stress and sowing times. Among different pollinator species, *A. mellifera* was found to be most abundant in sunflower. The visitation rate of pollinators was also higher in early-sown crop in the month of January as compared to sunflower crop sown in later months. Flowers from the well-watered crop had higher bee abundance and visit duration. Seed parameters were also affected by water deprivation and pollinator behavior. These findings indicate a complex interplay between the abiotic elements, plant–pollinator interactions, and crop productivity.

Our research found that overall bee abundance almost doubled in well-watered sunflower as compared to water-stressed treatments. Water limitation affected plant reproduction not only due to water stress but also because of the role played by pollinators, because there were fewer insects visiting the water-deprived plants. Specialist bees (*Eucera* sp.) have previously been reported to visit flowers independent of water stress, while generalist honey bees were found to prefer unstressed plant flowers [23]. The bottom-up effects of water shortage are more prominent in those plants sensitive to water stress, ultimately affecting floral resource production, which could ultimately impact specialist pollinator species [21,33,34,35]. Generalists like *A. mellifera*, due to their higher visit frequency, can dominate numerical abundance and high floral visitation frequency [36], and under resource limitation (i.e., nectar limitation caused by plants experiencing water stress) this may lead to honey bees outcompeting native bees [37]. Previous research has reported that drought conditions and reduced floral resource production lead to honey bees outcompeting bumble bees [38]. However, other bee species, like *Xylocopa* sp., could be more efficient in terms of pollination effectiveness parameters, as found in our study. The effectiveness of *Xylocopa* sp. has also been reported in various agricultural crops [39], and therefore could be considered as a potential solitary bee pollinator in sunflower.

Changes in temperature could directly affect plant–pollinator interactions. Warmer temperatures can reduce the lifespan of flowers, limiting the duration during which they offer resources to pollinators [40]. In our study, induced water stress significantly decreased pollinator abundance, which is similar to other evidence reporting fewer pollinators under drought conditions visiting wild mustard *Sinapis arvensis* L. (Brassicaceae) [41] and rapeseed *Brassica napus* L. (Brassicaceae) [42], mainly due to smaller flowers with less nectar. Previous studies also revealed that high temperatures can affect various crop growth stages such as seed setting and seed quality in peas and fava beans [43]. Moreover, temperature and water stress together have negative additive effects on nectar sugar concentration [21]. Other studies have revealed that as temperatures rise, the visitation rate of bees to the flower decreases [44]. Stress like drought can cause diverse effects for plant growth and development [45]. A previous study on pollination of *Trigonella moabitica* (Fabaceae) showed low visitation of honey bees on water-stressed plants, which was suggested to be due to the low attraction of the honey bees towards the reduced water and food-based signals [46].

In our study, January-sown sunflower showed higher yield traits. The results are similar to the study where low temperatures provided maximum floral resources for insect pollinators, particularly *A. mellifera*, for over two months, leading to a two to 29-fold increase in seed yield of oilseed echium *Echium plantagineum* L. [47]. A rise in temperature above 30 °C during the day and 20 °C at night directly decreases pollen availability, which may also result in lower yield production [48]. Plant activities such as seed germination, growth, development, photosynthesis, and reproduction are significantly affected by heat stress, which can have severe effects on plant growth, and hence affect the yield of the crop [49,50]. In sunflowers, heat stress during reproductive stages negatively affects fertilization, the quantity and rate of grain filling, embryo growth, seed weight, and the properties of the seed oil [51]. According to another study, there could be a 1.2% decrease in the final grain weight for every one-degree rise above 25 °C during the grain-filling stage of the crop [52]. In response to floral abundance, bees and other pollinators modify their foraging behavior (stay duration and visitation rate) and prefer to visit flowers that provide higher nectar [53]. Higher pollen deposition on the stigma of Brassica species due to these frequent visits leads to a higher yield [54].

Water stress causes a reduction in the nectar volume and sugar concentration [55,56], however this varies between species. A study noted that *Lathyrus pratensis*, *Onobrychis viciifolia*, and *Prunella vulgaris* (all Fabaceae) did not exhibit a decrease in nectar proportion per flower, mainly due to the drought resistance traits of these plant species [34]. Another study reported that in *Arabidopsis thaliana*, flower production recovered after an initial slow rate of new flower development when water stress was applied over many weeks [57]. Detailed studies revealed that water stress affects the metabolic transport of sucrose, which lowers the amount of starch and lipid stored in pollen grain [58]. Flower ovaries under drought stress have been reported to abort in a number of studies [59,60]. In the lentil, *Lens culinaris* Medik. (Fabaceae), post-fertilization abortion acted as a barrier to seed development even when pollen tubes reached the ovules and fertilization occurred [61]. Together, this suggested water stress ultimately leads to changes in flower traits and decreases in production.

Since water stress changes the assemblage of insect pollinators, future studies should evaluate whether drought-affected plants are visited by less efficient pollinators, leading to reduced crop reproductive success. One of the limitations of our study is that we evaluated the effect of drought and heat stress on sunflower reproductive success on a small scale. However, future studies should target large-scale studies and determine spatiotemporal mismatches between plants and their pollinators which will help to better understand threats to ecosystem conservation and food security. The impacts of drought on plant–pollinator interactions may pose challenges for food security, as insect pollination benefits over 75% of all crop species used for human consumption globally [12]. Moreover, from 1961 to 2016, the percentage of total agricultural land occupied by crops dependent on insect pollinators has increased regularly [62]. Pollinator reduction also threatens wild, non-crop plants. Pollinators carry forward plant communities by producing fruit and seeds, which conserve a significant part of biodiversity and endangered species [63,64,65]. This ecosystem service is essential for the conservation of biodiversity [66,67]. 

Another limitation of our study was our focus on one plant species. Future studies should look at a greater range of plant species and evaluate direct effects of drought on competition among pollinators collecting floral rewards. Moreover, further studies should focus on how drought stress affects plant reproduction on evolutionary time scales. Such comparisons could be expanded to other systems with several generalist and specialist pollinators in future research. More investigation is required to understand how the level of specialization affects environmental stress and behavioral interactions between plants and other animal pollinators. In the current study, we did not assess nocturnal pollination. Future studies could examine nighttime pollinators and their behavior in relation to water stress. In the current study, we did not identify the mechanisms underlying plant–pollinator interactions under water-stressed conditions. Furthermore, the effects of water stress on nectar production and sugar content were not covered in the current study. Long term studies on water stress and heat effects are needed to understand their effect on plant–pollinator interactions. Future studies should investigate the relative effects of drought on different floral signals and pollinator attractiveness.

## 5. Conclusions

Our study shows that the abundance and the visitation behavior of the pollinators were affected by water-stress as well as sowing phenology in sunflower, which may further affect the sunflower seed yield. We found a declining bee activity in April-sown sunflower. Moreover, pollinators preferred the well-watered treatments. The European honey bee, *Apis mellifera*, was the most abundant pollinator species, followed by the dwarf honey bee *A. florea*. A large carpenter bee species, *Xylocopa* sp., showed the highest visitation rate on sunflower florets, while the hover fly *Eristalinus aeneus* exhibited the longest visit duration. These findings show the complex relationship between insect pollinators, planting practices, and seed yield, which further emphasizes the need for pollinator and crop management to enhance the sunflower seed yield. Further research is needed to understand plant–pollinator interactions under water-stress conditions on different crops, to better formulate future strategies for years with higher weather extremes, as predicted under climate change.

## Figures and Tables

**Table 1:** Two-way effect of sowing month and water condition provided on abundance, visit duration and visitation rate of bee species.

Sowing	Water Regime	Abundance	Visit Duration	Visitation Rate (Floret)	Visitation Rate (Flower)
January	Water-deprived	4.85 ± 0.37 bc	0.64 ± 0.14	11.44 ± 0.63 a	1.69 ± 0.09 a
	Well-watered	2.34 ± 0.18 b	0.85 ± 0.14	10.19 ± 0.58 ab	1.57 ± 0.09 a
February	Water-deprived	1.01 ± 0.15 cd	11.30 ± 3.07	7.53 ± 0.60 c	1.43 ± 0.08 a
	Well-watered	2.22 ± 0.25 b	14.47± 3.72	10.00 ± 0.53 ab	1.67 ± 0.08 a
March	Water-deprived	1.50 ± 0.20 bc	12.67 ± 2.88	9.00 ± 0.70 bc	1.20 ± 0.05 b
	Well-watered	2.07 ± 0.28 a	9.04 ± 1.95	10.91 ± 0.65 a	1.47 ± 0.09 ab
April	Water-deprived	0.12 ± 0.05 d	12.58 ± 3.26	3.41 ± 0.49 d	0.64 ± 0.07 c
	Well-watered	0.43 ± 0.08 d	19.00 ± 7.59	4.24 ± 0.55 d	1.09 ± 0.14 c
	*F*, df	5.45, 3	1.06, 3	12.4, 3	2.64, 3
	*p*	0.0010	0.3644	<0.001	0.0490

Different letters indicate significant differences between treatments based on the LSD test at a 5% significance level.

**Table 2:** Effect of sowing month and pollinator species on abundance, visit duration and visitation rate of pollinator species.

Sowing Month	Pollinator Species	Abundance	Visit Duration	Visitation Rate (Floret)	Visitation Rate (Flower)
January	*A. dorsata*	1.84 ± 0.25 b	6.20 ± 1.22 bc	11.25 ± 0.56 bcde	2.25 ± 0.18 ab
	*A. florea*	1.98 ± 0.49 b	4.95 ± 1.13 bc	10.40 ± 0.51 cdef	1.35 ± 0.11 def
	*A. mellifera*	8.08 ± 0.55 a	3.10 ± 0.48 bc	11.55 ± 0.81 bcde	1.25 ± 0.12 def
	*E. aeneus*	0.56 ± 0.23 b	49.10 ± 8.78 a	2.05 ± 0.20 lm	1.15 ± 0.08 def
	*Halictus* sp.	0.24 ± 0.07 b	3.54 ± 0.49 bc	10.55 ± 0.53 bcde	1.65 ± 0.17 bcdef
	*Lasioglossum* sp.	0.66 ± 0.15 b	3.37 ± 0.51 bc	14.15 ± 0.89 abc	1.55 ± 0.15 cdef
	*Xylocopa* sp.	0.18 ± 0.05 b	5.73 ± 0.77 bc	15.75 ± 1.13 a	2.20 ± 0.14 ab
February	*A. dorsata*	0.77 ± 0.18 b	3.71 ± 0.53 bc	9.80 ± 0.62 defg	1.60 ± 0.15 bcdef
	*A. florea*	0.82 ± 0.16 b	2.84 ± 0.52 bc	8.10 ± 0.55 efgh	1.30 ± 0.13 def
	*A. mellifera*	7.55 ± 0.49 a	3.66 ± 0.66 bc	9.50 ± 0.96 efgh	1.50 ± 0.17 cdef
	*E. aeneus*	0.92 ± 0.21 b	71.93 ± 10.09 a	2.30 ± 0.24 klm	1.25 ± 0.12 def
	*Halictus* sp.	0.52 ± 0.11 b	3.03 ± 0.38 bc	7.90 ± 0.79 fgh	1.45 ± 0.14 cdef
	*Lasioglossum* sp.	0.55 ± 0.13 b	2.35 ± 0.40 bc	8.60 ± 0.86 efgh	1.70 ± 0.18 bcde
	*Xylocopa*	0.20 ± 0.06 b	2.69 ± 0.45 bc	15.15 ± 1.09 ab	2.05 ± 0.11 abc
March	*A. dorsata*	0.95 ± 0.23 b	2.93 ± 0.31 bc	16.90 ± 1.04 a	1.30 ± 0.13 def
	*A. florea*	7.63 ± 1.05 a	3.91 ± 0.51 bc	13.00 ± 0.68	1.20 ± 0.09 def
	*A. mellifera*	9.18 ± 0.60 a	3.11 ± 0.34 bc	13.35 ± 0.81 abcd	1.15 ± 0.08 def
	*E. aeneus*	0.02 ± 0.02 b	59.66 ± 8.65 a	1.80 ± 0.16 mn	1.10 ± 0.07 ef
	*Halictus* sp.	0.08 ± 0.04 b	2.18 ± 0.37 b	6.90 ± 0.81 ghij	1.30 ± 0.13 def
	*Lasioglossum*	0.40 ± 0.11 b	1.53 ± 0.35 c	7.55 ± 0.73 fghi	1.55 ± 0.17 cdef
	*Xylocopa* sp.	0.23 ± 0.08 b	2.09 ± 0.46 c	10.20 ± 0.83 def	1.75 ± 0.19 bcd
April	*A. dorsata*	0.33 ± 0.10 b	0.86 ± 0.24 c	6.35 ± 0.61 hij	1.20 ± 0.09 def
	*A. florea*	0.17 ± 0.08 b	1.06 ± 0.22 c	4.50 ± 0.46 jkl	1.05 ± 0.05 f
	*A. mellifera*	0.00 ± 0.00 b	0.00 ± 0.00 c	0.00 ± 0.00 n	0.00 ± 0.00 g
	*E. aeneus*	0.00 ± 0.00 b	0.00 ± 0.00 c	0.00 ± 0.00 n	0.00 ± 0.00 g
	*Halictus* sp.	0.00 ± 0.00 b	0.00 ± 0.00 c	0.00 ± 0.00 n	0.00 ± 0.00 g
	*Lasioglossum* sp.	0.10 ± 0.06 b	0.47 ± 0.12 c	4.65 ± 0.49 ijk	1.15 ± 0.08 def
	*Xylocopa* sp.	1.20 ± 0.21 b	2.84 ± 0.30 c	11.30 ± 0.71 bcde	2.65 ± 0.11 a
	*F*, df	30.85, 18	12.19, 18	20.99, 18	12.57, 18
	*p*	<0.001	<0.001	<0.001	<0.001

Different letters indicate significant differences between treatments based on the LSD test at a 5% significance level.

**Table 3:** Water regimes effect on bee species abundance, visit duration and visitation rate.

Bee Species	Water Regime	Abundance	Visit Duration	Visitation Rate (Floret)	Visitation Rate (Flower)
*A. dorsata*	Water-deprived	0.70 ± 0.12 c	2.61 ± 0.38 b	10.15 ± 0.80 bc	1.63 ± 0.12
	Well-watered	1.35 ± 0.19 c	4.24 ± 0.62 b	12.00 ± 0.76 ab	1.55 ± 0.11
*A. florea*	Water-deprived	1.94 ± 0.32 c	3.52 ± 0.62 b	9.13 ± 0.74 cde	1.13 ± 0.05
	Well-watered	4.17 ± 0.73 b	2.86 ± 0.29 b	8.88 ± 0.50 cde	1.33 ± 0.08
*A. mellifera*	Water-deprived	5.17 ± 0.38 b	2.43 ± 0.34 b	7.78 ± 0.93 e	1.03 ± 0.13
	Well-watered	8.95 ± 0.51 a	2.50 ± 0.38 b	9.43 ± 1.00 cde	0.93 ± 0.11
*E. aeneus*	Water-deprived	0.19 ± 0.05 c	49.88 ± 6.49 a	1.60 ± 0.19 g	0.88 ± 0.10
	Well-watered	0.65 ± 0.17 c	40.47 ± 6.67 a	1.48 ± 0.19 g	0.88 ± 0.10
*Halictus* sp.	Water-deprived	0.10 ± 0.03 c	1.76 ± 0.24 b	5.15 ± 0.67 f	1.03 ± 0.13
	Well-watered	0.38 ± 0.07 c	2.61 ± 0.36 b	7.53 ± 0.80 e	1.18 ± 0.14
*Lasioglossum* sp.	Water-deprived	0.30 ± 0.06 c	1.60 ± 0.25 b	8.18 ± 0.90 de	1.53 ± 0.11
	Well-watered	0.63 ± 0.11 c	2.26 ± 0.32 b	9.30 ± 0.58 cd	1.45 ± 0.11
*Xylocopa* sp.	Water-deprived	0.17 ± 0.04 c	3.28 ± 0.44 b	12.95 ± 0.89 a	2.00 ± 0.08
	Well-watered	0.54 ± 0.09 c	3.40 ± 0.35 b	13.25 ± 0.63 a	2.33 ± 0.13
	*F*, df	16.68, 6	2.89, 6	2.72, 6	1.69, 6
	*p*	<0.001	0.0089	0.0129	0.1216

Different letters indicate significant differences between treatments based on the LSD test at a 5% significance level.

**Table 4:** Effect of sowing month and water regime on flower head diameter, head weight, seed number and seed weight.

Sowing Month	Water Level	Head Diameter	Head Weight (g)	Seed No.	Seed Weight (g)
January	Water-deprived	12.15 ± 0.64	47.53 ± 2.18	705.13 ± 41.35	25.23 ± 0.96
	Well-watered	12.94 ± 0.97	53.90 ± 5.52	825.37 ± 106.45	28.20 ± 3.33
February	Water-deprived	10.71 ± 0.22	45.49 ± 2.38	637.78 ± 25.73	23.98 ± 1.57
	Well-watered	10.94 ± 0.33	48.77 ± 3.48	733.13 ± 67.16	26.33 ± 2.24
March	Water-deprived	9.79 ± 0.28	39.26 ± 2.70	553.48 ± 44.22	19.95 ± 1.78
	Well-watered	10.56 ± 0.35	48.68 ± 4.85	725.33 ± 83.49	27.75 ± 3.43
April	Water-deprived	8.81 ± 0.21	32.62 ± 1.94	493.50 ± 29.82	17.65 ± 1.25
	Well-watered	8.78 ± 0.42	31.22 ± 3.62	498.72 ± 52.50	17.20 ± 2.10
	*F*, df	1.42, 3	0.87, 3	1.04, 3	1.02, 3
	*p*	0.2524	0.4652	0.3866	0.3944

**Table 5:** Water regime and pollination type effect on head diameter, head weight, seed number and seed weight.

Water Regime	Pollination treatment	Head Diameter	Head Weight (g)	No. Seeds	Seed Weight (g)
Water-deprived	Caged	10.06 ± 0.28	40.54 ± 2.31	557.01 ± 30.53	20.57 ± 1.99
	Open	10.67 ± 0.56	41.91 ± 2.37	637.94 ± 33.70	29.17 ± 1.92
Well-watered	Caged	10.02 ± 0.45	39.04 ± 3.54	567.83 ± 44.94	21.36 ± 1.45
	Open	11.59 ± 0.63	52.25 ± 3.31	823.44 ± 58.76	22.04 ± 1.18
	F, df	0.13, 1	1.55, 1	2.71, 1	2.14, 1
	*p*	0.7176	0.2216	0.1088	0.1526

**Table 6:** Effect of sowing month and pollination type on head diameter, head weight, seed number and seed weight.

Sowing	Pollination Treatment	Head Diameter (inches)	Head Weight (g)	No. Seeds	Seed Weight (g)
January	Caged	10.91 ± 0.46 b	44.10 ± 3.10	618.65 ± 45.04 bc	23.15 ± 1.88
	Open	14.19 ± 0.36 a	57.33 ± 3.48	911.85 ± 62.14 a	30.28 ± 2.06
February	Caged	10.64 ± 0.36 b	45.93 ± 3.74	630.30 ± 46.52 bc	24.17 ± 2.45
	Open	11.02 ± 0.15 b	48.33 ± 2.07	740.62 ± 51.96 ab	26.15 ± 1.29
March	Caged	10.00 ± 0.38 bc	39.29 ± 3.69	530.05 ± 50.90 c	20.20 ± 2.47
	Open	10.36 ± 0.32 b	48.65 ± 4.16	748.77 ± 67.11 ab	27.50 ± 3.10
April	Caged	8.62 ± 0.35 d	29.83 ± 3.26	470.68 ± 52.74 c	16.35 ± 1.93
	Open	8.97 ± 0.30 cd	34.00 ± 2.16	521.53 ± 24.62 c	18.50 ± 1.35
	*F*, df	12.48, 3	1.56, 3	3.53, 3	1.40, 3
	*p*	<0.001	0.2170	0.0248	0.2596

Different letters indicate significant differences between treatments based on the LSD test at a 5% significance level.

## Data Availability

The data that support the findings of this study are available on request from the corresponding author, M.A.
